# Human Papillomavirus Vaccination: Past, Present and Future

**DOI:** 10.3390/vaccines10091398

**Published:** 2022-08-26

**Authors:** Gulzhanat Aimagambetova, Azliyati Azizan

**Affiliations:** 1 Department of Biomedical Sciences, School of Medicine, Nazarbayev University, Nur-Sultan 010000, Kazakhstan; 2 Basic Sciences Department, College of Osteopathic Medicine, Touro University Nevada, Henderson, NV 89014, USA

The link between human papillomavirus (HPV) infection and different diseases has been well-established since more than four decades ago [1,2]. Moreover, high-risk HPV types were confirmed as causative agents for a variety of malignant conditions, including oropharyngeal, anogenital, and cervical cancers [2].

Fortunately, for the past 15 years, there have been HPV vaccines available as a primary preventative measure and approved in many countries worldwide to prevent HPV-related diseases [3]. Having been approved since 2006, the HPV vaccines, without reservation, proved their effectiveness in the prevention of HPV-related cancers and benign conditions [4,5].

HPV vaccination is recommended before sexual debut for both girls and boys [3,4,5]. However, optional regimens that allow vaccination in older age groups have also been approved [5,6]. HPV vaccination is now successfully implemented in many countries; the USA and UK, Australia, and Argentina [4,5,7,8,9]. Successful implementation of HPV vaccination campaigns at the National levels in these countries has significantly reduced HPV-related cancers’ incidence and prevalence [10,11].

However, in spite of the aforementioned successes, the outcomes of the HPV vaccination campaigns are not similar worldwide. There are many reports that provide examples of the HPV vaccination program’s failure [12,13,14,15]. There are multiple reasons behind this; these include low public knowledge and awareness of HPV and related conditions, lack of educational campaigns, and poor vaccination program implementation plan [15]. Many countries report negative attitudes toward the vaccination even when the HPV vaccination program has been implemented, leading to low acceptance and coverage [13,15]. Furthermore, the HPV vaccination coverage and access are not equal in high- and low-middle-income countries [16,17]. Even developed countries experience HPV vaccination coverage disparities [18]. In addition, this inequality was aggravated by the COVID-19 pandemic impact on vaccination uptake in general and on HPV vaccination in particular [11].

All pieces of evidence mentioned above suggest that there is an emerging need to reassess all factors leading to HPV vaccination campaign failures on country-specific local levels. A deep investigation into the problem will help to develop an effective vaccine implementation plan, which should consider all interfering points [19]. The overall goal worldwide is to eliminate HPV-related diseases by increasing HPV vaccination coverage [16] and improving HPV vaccination knowledge and awareness. All these efforts consequently should positively affect the populations’ attitudes towards HPV vaccination and increase its acceptance. Effective context-specific interventions to improve the HPV vaccination public acceptance should be developed locally and worldwide [15].

In this context, the articles published in this Special Issue will represent the significant step forward necessary to stimulate further discussions among potential readers and experts in the field. Collectively, all of the published articles will serve as instruments to facilitate our joint efforts on the way forward to affect important changes in the HPV vaccination practice, and mobilize potential positive paradigm shifts in the future development of HPV immunization.
